# Human multipotent adult progenitor cells enhance islet function and revascularisation when co-transplanted as a composite pellet in a mouse model of diabetes

**DOI:** 10.1007/s00125-016-4120-3

**Published:** 2016-10-04

**Authors:** João Paulo M. C. M. Cunha, Gunter Leuckx, Peter Sterkendries, Hannelie Korf, Gabriela Bomfim-Ferreira, Lutgart Overbergh, Bart Vaes, Harry Heimberg, Conny Gysemans, Chantal Mathieu

**Affiliations:** 1 Laboratory of Clinical and Experimental Endocrinology, Katholieke Universiteit Leuven (KULEUVEN), Campus Gasthuisberg O&N1, Herestraat 49 bus 902, 3000 Leuven, Belgium; 2 Beta cell neogenesis laboratory, Diabetes Research Center, Vrije Universiteit Brussel, Brussels, Belgium; 3 ReGenesys BVBA, Heverlee, Belgium

**Keywords:** Islet, Revascularisation, Stem cells, Type 1 diabetes

## Abstract

**Aims/hypothesis:**

Hypoxia in the initial days after islet transplantation leads to considerable loss of islet mass and contributes to disappointing outcomes in the clinical setting. The aim of the present study was to investigate whether co-transplantation of human non-endothelial bone marrow-derived multipotent adult progenitor cells (MAPCs), which are non-immunogenic and can secrete angiogenic growth factors during the initial days after implantation, could improve islet engraftment and survival.

**Methods:**

Islets (150) were co-transplanted, with or without human MAPCs (2.5 × 10^5^) as separate or composite pellets, under the kidney capsule of syngeneic alloxan-induced diabetic C57BL/6 mice. Blood glucose levels were frequently monitored and IPGTTs were carried out. Grafts and serum were harvested at 2 and 5 weeks after transplantation to assess outcome.

**Results:**

Human MAPCs produced high amounts of angiogenic growth factors, including vascular endothelial growth factor, in vitro and in vivo, as demonstrated by the induction of neo-angiogenesis in the chorioallantoic membrane assay. Islet–human MAPC co-transplantation as a composite pellet significantly improved the outcome of islet transplantation as measured by the initial glycaemic control, diabetes reversal rate, glucose tolerance and serum C-peptide concentration compared with the outcome following transplantation of islets alone. Histologically, a higher blood vessel area and density in addition to a higher vessel/islet ratio were detected in recipients of islet–human MAPC composites.

**Conclusions/interpretation:**

The present data suggest that co-transplantation of mouse pancreatic islets with human MAPCs, which secrete high amounts of angiogenic growth factors, enhance islet graft revascularisation and subsequently improve islet graft function.

**Electronic supplementary material:**

The online version of this article (doi:10.1007/s00125-016-4120-3) contains peer-reviewed but unedited supplementary material, which is available to authorised users.

## Introduction

To date, insulin therapy is considered the gold standard for the treatment of type 1 diabetes. Nevertheless limitations persist, such as frequent episodes of hypoglycaemia, and development chronic micro- and macrovascular complications [1, 2]. Islet transplantation offers an alternative treatment for patients with type 1 diabetes, especially for those with hypoglycaemic unawareness following insulin administration. Despite the improved outcome of islet transplantation over the last few years, drawbacks remain, such as a limited supply of cadaveric donors, the necessity of several donors for a single transplantation and (immediate) graft failure through metabolic pressure, continued autoimmunity, alloimmunity, high concentrations of immunosuppressive drugs and oxidative stress caused by hypoxia or due to cytokine exposure [3]. Rapid revascularisation of the islets seems crucial to avoid beta cell stress by hypoxia and inflammatory cytokines. Several studies aiming to improve engraftment with cell therapy are currently ongoing (Clinical Trial.gov registration no. NCT00646724, NCT02384018).

Mesenchymal stem cells (MSCs, 30–35 μm in diameter), the major stem cells used for cell therapy, are self-renewing cells that can be isolated from the bone marrow and possibly also from many (if not all) tissues; for over 10 years MSCs have shown benefits in the treatment of several clinical diseases, mainly tissue injury and immune disorders [4–6]. When co-transplanted with islets, MSCs improve islet transplantation outcome in preclinical animal models through immunomodulation, increasing islet revascularisation and/or preserving islet morphology [7–10]. Despite the progress made in the understanding of MSC biology, there is little knowledge on the nature of their local microenvironment (probably hypoxic) and their functions in vivo. Moreover, long population doubling time, early senescence and DNA damage during in vitro expansion, as well as poor engraftment after transplantation, are considered to be among the key disadvantages of MSC therapy [11]. Furthermore, with long-term culture expansion, MSCs can become karyotypical abnormal, potentially posing a risk of tumour formation.

Multipotent adult progenitor cells (MAPCs, 15–20 μm in diameter) are adult stem cells isolated from postnatal bone marrow, muscle or brain from rodents and humans [12, 13]. These non-haematopoietic, non-endothelial stem cells most probably use similar immunosuppressive and angiogenic mechanisms to those used by MSCs, while displaying unique features (e.g. secretome, transcriptome and microRNA profiles) distinct from those of most adult stem cells [14–17]. Interestingly, MAPCs have been shown to promote tissue repair and healing and induce neo-angiogenesis, possibly by delivering angiogenic growth factors that activate or recruit endogenous vascular cells and that seem to be specifically tailored to the immediate needs of the injured tissue [18–21]. In vivo, these cells are short-lived as they experience only a minimally prolonged residence time. Moreover, in contrast to other cell types, MAPCs can be expanded in the long term (for > 80 population doublings) without genetic instability and can be administered without tissue matching, making them into an optimal stem cell product for routine clinical use (MultiStem; Athersys, Cleveland, OH, USA). Therefore, we aimed in this study to assess the therapeutic efficacy of co-transplantation of undifferentiated human MAPCs with mouse islets as separate or composite pellets in a syngeneic marginal mass islet transplantation model.

## Methods

### Animals

C57BL/6 mice (purchased from Charles River, L’Arbresle, France) were used as islet donors and transplant recipients in all procedures. The mice were handled in accordance with the *Guide for the Care and Use of Laboratory Animals*, 18th edition (2011) as well as Katholieke Universiteit Leuven (KULEUVEN, Leuven, Belgium) Animal Care and Use Guidelines.

### Preparation and characterisation of human MAPCs

Human MAPCs (*n* = 2) used in this study were isolated by ReGenesys BVBA (Athersys affiliate; Heverlee, Belgium) from the bone marrow of a 30-year-old female and a 45-year-old male volunteer, with informed consent and ethical approval. Isolation and culture of the cells was carried out as outlined in electronic supplementary material (ESM) Methods [22].

Phenotypic analysis of the human MAPCs was performed using fluorochrome-conjugated antibodies recognising cluster of differentiation (CD) 3, CD31, CD34, CD40, CD44, CD86, CD105, fetal liver kinase 1 (Flk1), HLA-ABC and HLA-DR (ebioscience, San Diego, CA, USA). Acquisition was done by using a Gallios multicolour flow cytometer (Beckman Coulter, Suarlée, Belgium). For analysis of the samples, FlowJo version 10.1 (Tree Star, Ashland, OR, USA) software was used.

Cell-free supernatant fractions were assayed for a pro-inflammatory, cytokine, chemokine, angiogenesis and vascular inflammation panel (see ESM Methods) by multiplex electrochemiluminescence (Meso Scale Discovery, Rockville, MD, USA) as per manufacturer’s protocol.

The angiogenic potential of human MAPCs was examined in the chick chorioallantoic membrane (CAM) as described [23].

### Marginal mass syngeneic islet transplantation diabetes model

To induce diabetes in recipients, a single intravenous injection of alloxan (90 mg/kg body weight; Sigma-Aldrich, St. Louis, MO, USA) was administered to 8- to 10-week-old male C57BL/6 mice, and animals were considered to be diabetic after two consecutive non-fasting tail-vein blood glucose concentrations of ≥ 11.1 mmol/l, measured by an AccuChek Glucometer (Roche Diagnostics, Vilvoorde, Belgium). Before transplantation, islets from 2- to 3-week-old male C57BL/6 mice were isolated by collagenase digestion, washed, counted and sometimes mixed with human MAPCs (see ESM Methods) [24]. For more details see ESM Methods. On weeks 2 and 5 after transplantation, graft-bearing kidneys were removed and fixed in 4% formaldehyde followed by paraffin embedding or were used for RNA isolation.

### Physiological studies

Glucose tolerance tests were performed after mice had been fasted for 16 h. Mice were injected intraperitoneally with d-glucose (2 g/kg body weight), and blood glucose levels were measured at the indicated times.

For serum insulin and C-peptide determination, blood was collected from the saphenous vein. Serum was isolated by centrifugation and levels of pancreatic hormones were determined by ultrasensitive ELISA kits (Mercodia, Uppsala, Sweden; Merck Millipore, Billerica, MA, USA).

### Morphometry and immunohistochemistry

Graft-bearing kidneys were embedded in paraffin and 6 μm sections were obtained from the total graft area. Stainings for insulin (guinea pig, no. A0564; Dako Belgium, Heverlee, Belgium), glucagon (mouse, no. G2654; Sigma, St Louis, MO, USA), somatostatin (rat, no. ab30788; Abcam, Cambridge, UK), endomucin (rat, no. sc-65495; Santa Cruz Biotechnology, Santa Cruz, CA, USA) and α-smooth muscle actin (α-SMA) (goat, no. EB06450; Everest Biotech, Upper Heyford, UK) were used to evaluate beta cell mass and blood vessel density with the aid of a Ventana Ultra staining platform (Roche). The endomucin and α-SMA antibodies are recommended for detection of endomucin or α-SMA, respectively, of mouse and not human origin. All antibodies were validated in previous studies.

For quantification of beta cell and blood vessel volume, all images were captured using a Nikon Eclipse TE2000-E microscope using a × 40 magnification objective and the large image-capture feature so that the entire graft area of each section could be pictured at once. Insulin-, glucagon- and somatostatin-positive areas, as well as endomucin-positive areas, within the endocrine compartment of the islet graft were measured semi-automatically by open-source image processing package based on ImageJ, Fiji version software (National Institutes of Health, Bethesda, MD, USA) on approximately 15% (i.e. every fifth section) of the total graft as described [25]. The vessel/beta cell ratio was calculated as (blood vessel area/insulin-positive area) × 100%. Vessel density was calculated as the number of intra-islet vessels per mm^2^.

### Quantitative PCR

Islet graft RNA was isolated as described [26]. Primer and probe sequences are listed in ESM Table 1. Results are expressed as ${2}^{-\Delta \Delta {\mathrm{C}}_{\mathrm{t}}}$ (see ESM Methods).

### Statistics

Statistics were calculated with Prism software 5.0 (GraphPad Software, San Diego, CA, USA). The χ^2^ test was applied to identify the significance of the difference between diabetes reversal rates when comparing different groups. All numerical values were presented as the mean ± SEM, unless otherwise indicated. Significance was determined using the Mann–Whitney *U* test or Kruskal–Wallis test, and a value of *p* < 0.05 was considered significant.

## Results

### Human MAPCs secrete angiogenic growth factors and have neo-angiogenic potential in the in vivo CAM assay

**Fig. 1:**
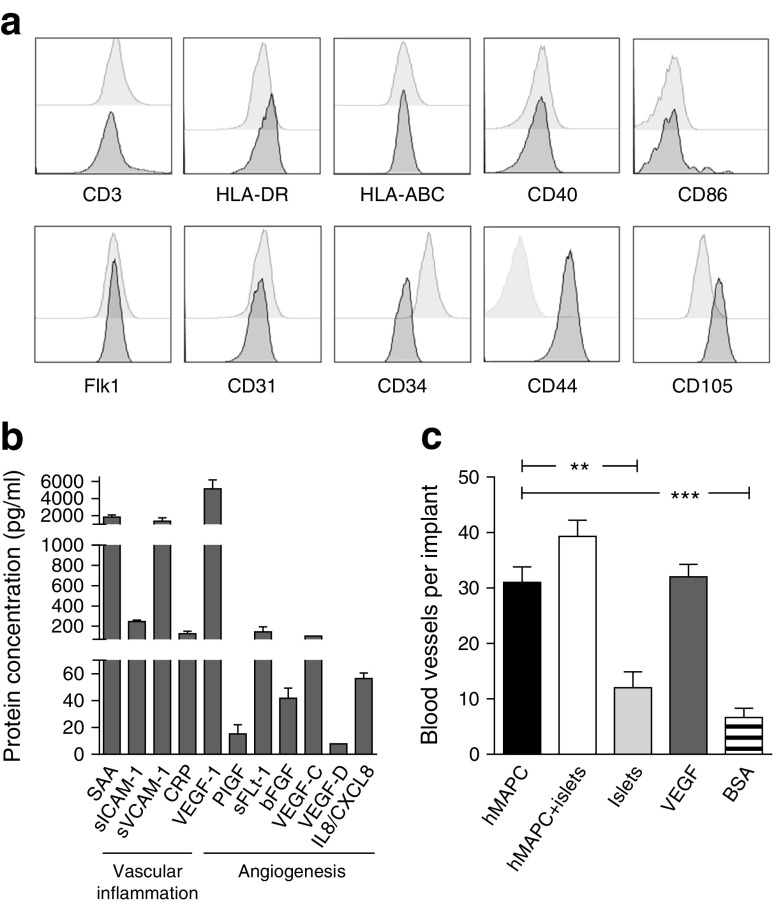
Characterisation of human MAPCs. (**a**) Cell surface marker expression of human bone marrow-derived MAPCs. Flow cytometry histograms show the expression levels (peaks shaded dark grey) of selected markers associated with the characterisation of human MAPCs (CD44 and CD105) compared with negative isotype controls (peaks shaded light grey). (**b**) Culture medium of human MAPCs was analysed with human biomarker 40-Plex kit containing a pro-inflammatory panel, cytokine panel, chemokine panel, angiogenesis panel and vascular inflammation panel. Data are means ± SEM. SAA, serum amyloid A; sVCAM-1, soluble vascular cell adhesion molecule-1; CRP, C-reactive protein. (**c**) Pro-angiogenic properties of 2.5 × 10^5^ human MAPCs (hMAPC) with or without 150 C57BL/6 mouse islets in a CAM assay; 150 C57BL/6 mouse islets alone and BSA were used as negative controls and VEGF-A as positive control (mean ± SEM, *n* = 3–9 per group). **p < 0.01 and ***p < 0.001 for indicated comparisons

Human MAPCs expressed low levels of HLA-ABC (<25%) and they did not express HLA-DR, CD40, CD86, CD3, Flk1 (also known as vascular endothelial growth factor [VEGF] receptor 2 or kinase insert domain receptor [KDR]), CD31 (also known as platelet endothelial cell adhesion molecule [PECAM]-1) or CD34 (<1%), which are typical cell surface markers for MHC class II and co-stimulation molecules, T cells and endothelial cells, respectively (Fig. 1a). Human MAPCs were positive for CD44 and CD105 (>95%) [27]. Their surface marker signature defines a unique phenotype that distinguishes them from any other known class of stem cells [27].

The culture supernatant fraction of human MAPCs was analysed with human biomarker 40-Plex kit containing a pro-inflammatory panel, cytokine panel, chemokine panel, angiogenesis panel and vascular inflammation panel (Fig. 1b). The cells produced numerous angiogenic growth factors, including VEGF (VEGF-A, -C and -D), placental growth factor (PlGF), soluble fms-like tyrosine kinase-1 (sFlt-1), basic fibroblast growth factor (bFGF) and IL8. On the other hand, the cells secreted negligible amounts of various cytokines (IFN-γ, IL1α, IL1β, IL2, IL4, IL5, IL6, IL7, IL10, IL12p70, IL12/IL23p40, IL13, IL15, IL16, IL17A, TNF-α and TNF-β) and chemokines (eotaxin, eotaxin-3, IFN-γ-induced protein 10 [IP-10], monocyte chemoattractant protein [MCP]-1, MCP-4, macrophage-derived chemokine [MDC], macrophage inflammatory protein [MIP]-1α, MIP-1β and thymus- and activation-regulated chemokine [TARC]) (data not shown).

The neo-angiogenic potential of human MAPCs was tested using the CAM angiogenesis model. Inoculation with 5 μg recombinant human VEGF markedly increased the number of blood vessels directed toward the implant (Fig. 1c). Human MAPCs (2.5 × 10^5^) significantly increased vessel formation by 2.5- and 4.6-fold compared with control implants containing either 150 C57BL/6 mouse islets or 50 μg BSA, respectively (Fig. 1c).

### Co-transplantation of islets–human MAPCs as a composite pellet improves the outcome of marginal mass islet transplantation

We titrated the number of pancreatic islets transplanted to determine ‘a marginal islet mass’ that would be just at the edge of achieving normoglycaemia in around 50% of recipients. Transplantation of 50 syngeneic C57BL/6 islets did not reverse hyperglycaemia (0 out of 7 mice), whereas 100% (4 out of 4 mice) achieved normoglycaemia when 300 islets were transplanted under the kidney capsule. We assessed that the marginal islet number was approximately 150 islets (25 out of 45 mice, 56% achieving normal blood glucose concentrations 5 weeks post-transplantation). This number of islets was selected for further experiments.

**Fig. 2:**
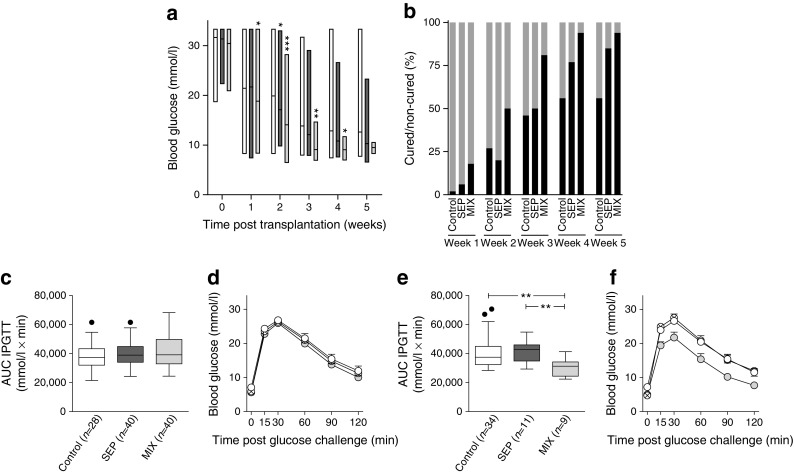
In vivo function of a marginal islet mass co-transplanted with human MAPCs. (**a**) Blood glucose measurements of alloxan-induced diabetic C57BL/6 mice transplanted with 150 islets alone (control [*n* = 47]; white bars) or with 150 islets co-transplanted as separate (*n* = 52; dark-grey bars) or composite pellets (*n* = 40; light-grey bars) with 2.5 × 10^5^ human MAPCs. Floating bars extend from the minimum to the maximum, the line indicates the mean.*p < 0.05, **p < 0.01 and ***p < 0.001 vs islets alone (control). (**b**) Percentage of cured (black bars) and non-cured (grey bars) mice after islet transplantation. (**c**–**f**) AUCs and blood glucose measurements after IPGTTs in mice transplanted with a marginal islet mass alone (control, white circles) or combined with human MAPCs as a separate (crossed circles) or composite pellet (grey circles) 2 (**c**, **d**) and 5 (**e**, **f**) weeks after transplantation. **p < 0.01 for indicated comparisons. The box extends from the 25th to 75th percentiles, the line indicates the median, and the Tukey method was used to plot the whiskers and the outliers (solid black dots); (**d**, **f**) mean ± SEM

Next, we investigated the outcome of the co-transplanted marginal islet mass with 2.5 × 10^5^ human MAPCs as separate or composite pellets and monitored the blood glucose levels of transplanted mice for up to 5 weeks. Co-transplantation of pancreatic islets with human MAPCs as separate pellets (SEP) slightly improved the average blood glucose concentrations compared with mice transplanted with islets alone. Interestingly, mice receiving islet–human MAPC composites (MIX) had better glycaemic control at all measured time points from 2 weeks onwards (Fig. 2a). Three weeks after transplantation, 81% of the MIX mice (13 out of 16 mice) were normoglycaemic compared with 50% of the SEP mice (13 out of 26 mice; *p* < 0.05) and 47% in the mice transplanted with islets alone (21 out of 45 mice in the control group; *p* < 0.05) (Fig. 2b). By the end of the observation period (week 5 post-transplantation), an even greater proportion of mice co-transplanted with islets–human MAPCs reversed diabetes compared with mice transplanted with islets alone (94% in the MIX group [*p* < 0.01] and 85% in the SEP group [*p* < 0.001] vs 56% in the control group). After nephrectomy, the blood glucose concentrations of normoglycaemic islet recipients rapidly progressed to severe hyperglycaemia, indicating that the improvement in metabolic glucose control had resulted from the transplanted syngeneic islets and not from the regeneration of remnant islets in the alloxan-treated pancreas of the islet recipients (ESM Fig. 1 a–c). Moreover, there was no significant difference in body weight between transplant recipients from different experimental groups on day 0 (22.8 ± 0.27 g, 23.5 ± 0.2 g and 22.9 ± 0.21 g for the control, SEP and MIX groups, respectively; *n* = 40–52) or at week 5 post-transplantation (25.6 ± 0.33 g, 26.2 ± 0.32 g and 25.4 ± 0.27 g, for the control, SEP and MIX groups, respectively; *n* = 40–52) (ESM Fig. 1d).

Serum insulin and C-peptide levels were measured at 2 and 5 weeks after transplantation, as an index of islet graft function. At week 2 post-transplantation, insulin and C-peptide concentrations did not differ significantly between the various experimental groups (ESM Fig. 2 a, b). However, at week 5 post-transplantation, C-peptide values were significantly higher in the SEP mice (304 ± 80 pmol/l; *n* = 10, *p* < 0.01) as well as in the MIX mice (282 ± 77 pmol/l in the MIX group; *n* = 10, *p* = 0.05) when compared with values from the mice transplanted with islets alone (232 ± 52 pmol/l in the control group; *n* = 10) (ESM Fig. 2 c, d).

To investigate the insulin secretory capacity of the islet transplant, a series of IPGTTs were performed at weeks 2 and 5 post-transplantation. At week 2 post-transplantation, there were no significant differences in glucose clearance among the studied groups (Fig. 2 c, d). At week 5 post-transplantation, however, MIX mice cleared glucose more efficiently than SEP mice or mice transplanted with islets alone (control) (Fig. 2 e, f). To further support the observations from the IPGTT, the AUC was calculated and found to differ significantly between the MIX group and the SEP group (*p* < 0.01) or the group transplanted with islets alone (control) (*p* < 0.01). (Fig. 2e).

### Increased beta and alpha cell volume and blood vessel formation in mice transplanted with islet–human MAPC composites

**Fig. 3:**
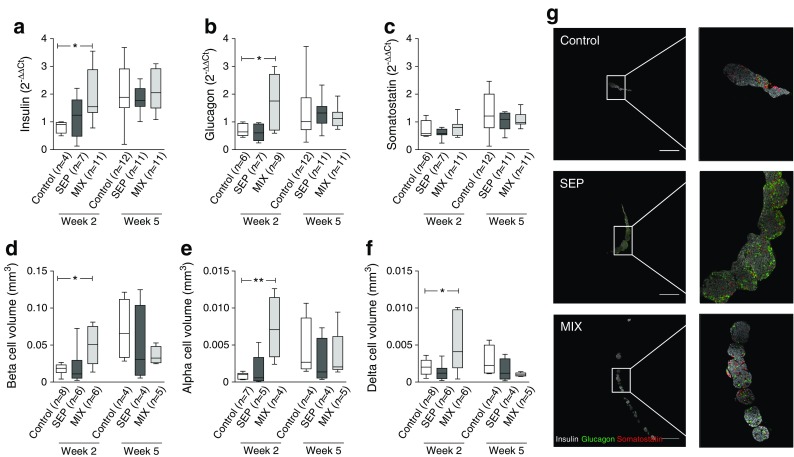
Morphology and composition of islets co-transplanted with human MAPCs examined at 2 and 5 weeks post-transplantation. (**a**–**c**) Box and whiskers plots of mRNA levels of mouse insulin, glucagon and somatostatin in isolated islet grafts derived from mice transplanted with a marginal islet mass alone (control) or combined with human MAPCs as a separate or composite pellet. Values were normalised to the geometric mean of housekeeping genes. Data are expressed as ${2}^{-\Delta \Delta {\mathrm{C}}_{\mathrm{t}}}$. The box extends from the 25th to 75th percentiles, the line indicates the median, and the Tukey method was used to plot the whiskers and the outliers. Statistical significance was calculated using Mann–Whitney *t* tests. *p < 0.05 for indicated comparisons. (**d**–**f**) Box and whiskers plots of volumes of beta, alpha and delta cells of grafts derived from mice transplanted with a marginal islet mass alone or combined with human MAPCs as a separate or composite pellet. The box extends from the 25th to 75th percentiles, the line indicates the median, and the Tukey method was used to plot the whiskers and the outliers. Statistical significance was calculated using Mann–Whitney *t* tests. *p < 0.05 and **p < 0.01 for indicated comparisons. (**g**) Distribution of mouse insulin- (white), glucagon- (red), and somatostatin (green)-positive cells in islet grafts composed of islets and human MAPCs as separate (SEP) or composite (MIX) pellets or of islets alone (control) at 2 weeks post-transplantation. Stitched composite images are representative of sections from 4–8 different mice. Scale bar, 100 μm. Higher magnification of the boxed area in control demonstrates a compact graft with normal distribution of insulin, glucagon and somatostatin positivity. Higher magnification of the boxed area in the SEP and MIX groups shows spread-out grafts with high insulin and glucagon positivity

Grafts from the three groups of mice were evaluated for their gene profile, cytoarchitecture and revascularisation process. Insulin and glucagon mRNA expression levels were significantly higher in MIX mice than in the mice transplanted with islets alone (control) 2 weeks after transplantation (Fig. 3 a, b). There was no difference in somatostatin mRNA expression levels at this time point (Fig. 3c). At week 5 post-transplantation, the intra-graft mRNA levels of the studied endocrine hormones were similar in all groups. These measures were corroborated by histological analysis of the grafts, which showed significantly higher insulin-, glucagon- and somatostatin-positive areas in the grafts of MIX mice 2 weeks after transplantation when compared with grafts from mice transplanted with islets only (control) (Fig. 3 d–g).

**Fig. 4:**
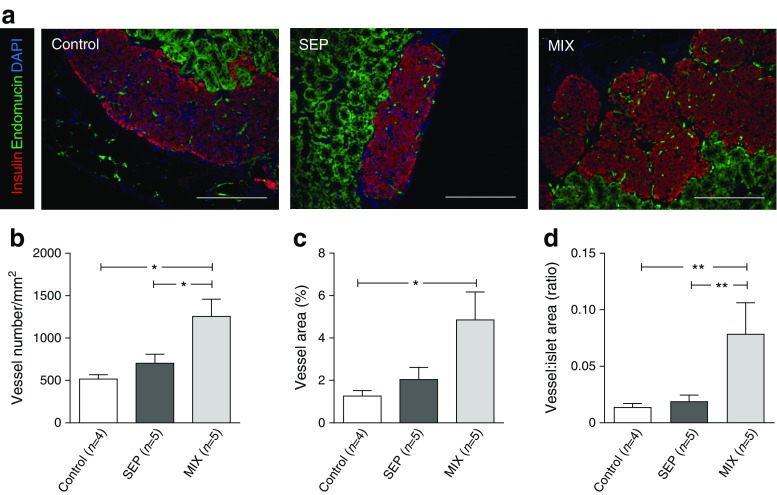
Co-transplantation of islets with human MAPCs as composites promotes graft revascularisation in a marginal islet mass diabetic mouse model. (**a**) Representative sections of 5 week grafts consisting of mouse islets transplanted alone (control) or with human MAPCs as separate or composite pellets. Images are representative of insulin and endomucin (vessel) staining for 3 or 4 mice in each transplant group. Scale bar, 100 μm. (**b**–**d**) Assessment of vessel morphological variables was carried out as described in the Methods. Data are means ± SEM. Statistical analysis was performed using Mann–Whitney *t* tests. *p < 0.05 and **p < 0.01 for indicated comparisons

The formation of blood vessels was assessed by measuring the expression of endomucin, a marker for vascular endothelial cells. At week 2 post-transplantation, graft vessel density and area, as well as the ratio of the vessel area to insulin-positive area, did not differ between the studied groups (data not shown). At week 5 post-transplantation, enhanced graft revascularisation was observed in MIX mice compared with SEP mice or mice transplanted with islets alone (control) (Fig. 4a). In the MIX grafts, 1256 ± 203 vessels/mm^2^ were detected compared with 702 ± 106/mm^2^ in the SEP grafts and 515 ± 52/mm^2^ in the islet-alone grafts (both *p* < 0.05; Fig. 4b). Another index of neo-angiogenesis, graft vessel area, was significantly higher in the MIX grafts (4.85 ± 1.32%, *n* = 5) than in the grafts of islets only (1.26 ± 0.25%; *n* = 4, *p* < 0.05, Fig. 4c). Additionally, mice transplanted with the MIX grafts had a higher ratio of vessel per insulin-positive area than either the SEP grafts or the islet-alone grafts (0.079 ± 0.027 vs 0.019 ± 0.006 and 0.014 ± 0.003 vessels per islet, both *p* < 0.01) (Fig. 4d). Collectively, grafts composed of islets mixed with human MAPCs retrieved 5 weeks after transplantation contained high numbers of endomucin-positive endothelial cells (Fig. 4a) and only a few α-SMA-positive cells (ESM Fig. 3a), indicating the presence of new capillary-like structures. Moreover, PCR analyses demonstrated that both mouse *Cd31* (*Pecam1*) and mouse *α-SMA* (*Acta2*) mRNA were more abundantly present in the MIX grafts than in the grafts of islets alone (ESM Fig. 3 b, c), while expression of the basement membrane marker *Col4a1* did not differ among the experimental groups (data not shown).

## Discussion

Several hurdles still prevent the progression of clinical islet transplantation, including early graft failure and the loss of transplanted islet mass due to non-immunological reasons [28]. In the first days following transplantation, islets lack an adequate vascular network, leading to severe hypoxia and cell death. It is believed that this is one of the major causes for the poor performance of islet grafts long-term. Therefore, there is a need of methods to improve the early survival, function and engraftment of transplanted islets. A variety of (stem) cell populations (i.e. endothelial progenitor cells, MSCs) have been described for enhancing transplanted beta cell survival and function after co-transplantation; however, there are still a number of problems relating to their wide-scale application in the clinic, such as inconsistent stem cell potency, poor cell engraftment and survival and age/disease-related host tissue impairment [29].

MAPCs are a novel class of progenitor cells and can be derived from the postnatal bone marrow stroma compartment and also from muscle and brain of several species, including rodents and humans [13]. These cells have demonstrated extensive in vitro expansion capacity, durable cytogenetic stability and higher plasticity when compared with MSCs [30]. MAPCs are also non-immunogenic and have a strong immunomodulatory profile [15, 27, 31–35], permitting safe non-HLA-matched allogeneic and even xenogeneic use without the need for immunosuppression [36, 37]. Interestingly, MAPCs seemed to exhibit an unusual capacity to evade the immune system and can regulate homeostatic T cell proliferation, prevent the expansion of T helper (Th) 1, Th17 and Th22 effector T cells and block the development of pathogenic allogeneic responses [31]. Moreover, MAPCs have been reported to secrete angiogenic growth factors and to improve vascular remodelling in different models of ischaemia, such as those for cardiac infarction and severe limb ischaemia [20, 37]. Based on these phenotypic and functional characteristics, we wanted to study the localised effect of human MAPCs on islet graft function in a murine syngeneic marginal mass transplant model.

Here we show for the first time that human MAPCs co-transplanted as composite pellets with mouse islets can improve islet graft function as measured by the initial glycaemic control, diabetes reversal rate, glucose tolerance and serum C-peptide concentration when compared with transplantation of islets alone. Moreover, we found that grafts composed of islet–human MAPC composites had an improved revascularisation process. The human MAPCs actively participated in the revascularisation process mainly by producing angiogenic growth factors, soluble adhesion molecules and IL8.

Several studies have shown that MAPCs from both allogeneic and xenogeneic sources exert positive effects in models of ischaemia, mainly through the secretion of trophic factors such as VEGF-A, platelet-derived growth factor and IGF-1 [37, 38]. Our present data corroborated the angiogenic potency of human MAPCs, as supernatant fractions of cultured cells were shown to contain high concentrations of vascular inflammation and angiogenic factors such as VEGF-A, PlGF and bFGF, all major regulators of islet vascularisation. The neo-angiogenic potential of human MAPCs was further validated in vivo in a chicken CAM assay, where implanted human MAPCs formed new blood vessels at a rate comparable with that observed with recombinant VEGF-A. VEGF-A is considered to be one of the major regulators of islet vascularisation, innervation and function, as beta cell-specific deletion of VEGF-A leads to diminished and abnormal islet vascularity, impaired postnatal nerve fibre ingrowth and dysregulated glucose-stimulated insulin secretion [39–41]. Moreover, VEGF-A seems to be required for revascularisation of transplanted islets. Based on these data, several investigators have tried to enhance VEGF production locally in the pancreatic islets, although not continuously as this might trigger vascular tumour formation [42–44]. It has been reported that islets co-transplanted with human embryonic stem cell-derived MSCs that conditionally overexpress VEGF allow a 50% reduction in the islet mass required to reverse diabetes in mice. These cells significantly improved islet metabolic function and revascularisation [10].

Islet engraftment is a slow process, and while vascular sprouting, angiogenesis and revascularisation occur within 1–2 weeks after transplantation, the maturation of the blood vessels is likely to take several weeks to even months. Although VEGF can boost the process of islet revascularisation, this protein is also critical for maintenance of the intra-islet endothelial cell pool [40]. We found a significant improvement in the development of a new islet capillary network in mice where islets were co-transplanted with islet–human MAPCs. Indeed, higher numbers of capillary-like structures with a lining of endothelial cells (detected with mouse-specific endomucin antibody) were found on the periphery and in the intra-islet space of the islet–human MAPC composites at week 5 post-transplantation. This suggests that host-derived vessels are directly feeding transplanted islets and that close proximity and even direct contact between the transplanted pancreatic islets and human MAPCs is of critical importance for the improved glucose control, diabetes reversal rate and increased revascularisation. Absence of human *Cd31* (*Pecam1*) mRNA expression in the islet grafts further supports the idea that human MAPCs are not incorporated into new capillaries but possibly secrete growth factors to initiate angiogenesis and to support functional tissue survival (data not shown). These observations are in full agreement with previous observations that the major role of human MAPCs is to provide angiogenic growth factors in the first days after implantation, after which they are cleared rapidly from the body, without leading to immune activation [18, 37].

The present data encouraged the use of human MAPCs in islet transplantation protocols as our results demonstrate the improvement of islet graft morphology and function by transplantation of islet–human MAPC composites, possibly via the promotion of graft revascularisation mediated by human MAPCs.

## Electronic supplementary material

Below is the link to the electronic supplementary material.

ESM (PDF 1618 kb)

## Abbreviations

α-SMA: α-Smooth muscle actin
bFGF: Basic fibroblast growth factor
CAM: Chorioallantoic membrane
CD: Cluster of differentiation
Flk1: Fetal liver kinase 1
KDR: Kinase inert domain receptor
MAPC: Multipotent adult progenitor cell
MSC: Mesenchymal stem cell
MIX: Mice transplanted with islets and human MAPCs as composite pellets
PECAM-1: Platelet endothelial cell adhesion molecule
PlGF: Placental growth factor
sFlt-1: Soluble fms-like tyrosine kinase-1
Th: T helper
SEP: Mice transplanted with islets and human MAPCs as separate pellets
VEGF: Vascular endothelial growth factor
