# Supplementary material for: Human multipotent adult progenitor cells enhance islet function and revascularisation when co-transplanted as a composite pellet in a mouse model of diabetes
Source: Diabetologia. 2016 Oct 4;60(1):134–42. doi: 10.1007/s00125-016-4120-3 (PMC6518081; doi:10.1007/s00125-016-4120-3)
Supplement: Supplementary file 1 — (PDF 1618 kb) [file 125_2016_4120_MOESM1_ESM.pdf]

## ESM Methods

### Preparation and characterization of human MAPC

Human MAPC (n=2) used in this study were isolated by ReGenesys BVBA (Athersys Inc. affiliate in Heverlee, Belgium) from bone marrow of a 30-year-old female and a 45-year-old male volunteer, with informed consent and ethical approval. Isolation and culture of the cells was carried out as described [1]. Human MAPC were expanded on the Quantum cell expansion system (Terumo BCT, Lakewood, CO). This closed automated culture system is comprised of a synthetic hollow-fibre bioreactor connected to sterile closed-loop, computer-controlled media and gas exchangers. The bioreactor contains ~11,000 fibres generating an expansion surface area of 2.1 m². After coating the bioreactor with fibronectin, cells were seeded on the inside of the hollow fibres and expanded in MAPC culture medium. Cells were harvested after 5-6 days using trypsin/EDTA.

Cell-free supernatants were assayed for human basic fibroblast growth factor (bFGF), C-reactive protein (CRP), eotaxin, eotaxin-3, soluble fms-like tyrosine kinase 1 (sFlt1), granulocyte-macrophage colony-stimulating factor (GM-CSF), soluble intracellular adhesion molecule-1 (sICAM-1), interferon-γ (IFN-γ), interleukin-1α (IL1α), IL1β, IL10, IL12 p70, IL12/IL23p40, IL13, IL15, IL16, IL17A, IL2, IL4, IL5, IL6, IL7, IL8, IFN-γ-induced protein-10 (IP-10), monocyte chemoattractant protein-1 (MCP-1), MCP-4, macrophage-derived chemokine (MDC), macrophage inflammatory protein-1α ( MIP-1α), MIP-1β, placental growth factor (PlGF), serum amyloid A (SAA), thymus- and activation-regulated chemokine (TARC), Tie2, tumour necrosis factor-α (TNF-α), TNF-β, soluble vascular cell adhesion molecule-1 (sVCAM-1), vascular endothelial growth factor-A (VEGF-A), VEGF-C, and VEGF-D by multiplex electrochemiluminescence (Meso Scale Discovery, Rockville, MD) as per manufacturer's protocol.

### Marginal mass syngeneic islet transplantation diabetes model

To induce diabetes in recipients, a single intravenous injection of alloxan (90 mg/kg; Sigma-Aldrich) was administered to male 8-10 week-old C57BL/6 mice (purchased from Charles River, L' Arbresle, France), and animals were considered to be diabetic after two consecutive non-fasting tail vein blood glucose concentrations of ≥11.1 mmol/l,

measured by an AccuCheck Glucometer (Roche Diagnostics Vilvoorde, Belgium). Before transplantation, islets from male 2-3 week-old C57BL/6 mice (purchased from Charles River, L' Arbresle, France) isolated by collagenase digestion were washed, counted, and in some cases mixed with human MAPC [2]. Thereafter, the cellular pellets were transferred to silicon microtubing (Becton Dickinson, Erembodegem, Belgium), centrifuged for 5 minutes at 1500 rpm. During transplantation, the mice were anaesthetized and the left kidney was exposed by a lumbar incision. Diabetic recipient mice were given 150 islets alone, 150 islets and $2.5 \times 10^5$ human MAPC as separate pellets (SEP) or 150 islets and $2.5 \times 10^5$ human MAPC as composite pellet (MIX) under the renal capsule. Mice were individually housed in filter-top cages, had *ad libitum* access to food pellets (Ssniff®, Bio Services BV, Uden, The Netherlands) and autoclaved water and received cage enrichment. Non-fasting blood glucose levels from the tail vein of each recipient were measured daily during the first week post-transplantation and thereafter three times weekly. Mice were considered cured when having blood glucose levels <11.1 mmol/l after 3 consecutive measurements. All islet transplantations were performed at random in all experimental groups. On week 2 and 5 after islet transplantation, graft-bearing kidneys were removed and fixed in 4% formaldehyde followed by paraffin embedding or were used for RNA isolation.

**Quantitative PCR**

Islet graft RNA was isolated as described [3], and a 1-µg aliquot was reverse transcribed into cDNA (Superscript II; Life Technologies, Carlsbad, CA). cDNA was then subjected to quantitative PCR using gene-specific forward and reverse primers (own design or purchased from Integrated DNA Technologies, Coralville, IA (CD31/PECAM-1) or Thermo Fisher Scientific, Waltham, MA (α-SMA)) using either Fast SYBR® Green Master Mix or a gene-specific TaqMan® probe in combination with TaqMan® Fast Universal Master Mix (Life Technologies). Primer and probes sequences are listed in ESM table 1. Each quantitative reaction was carried out in duplicate or triplicate, and islet grafts from 6-11 mice of each experimental group were independently tested. All samples were normalized to the average of Actine, HPRT and RPL27 as housekeeping genes. Background amounts of each target gene were calculated from the non-grafted kidney.

The data were analysed using the comparative Ct method, in which the amount of target, normalised to an endogenous reference gene (normalisation gene) and relative to a calibrator (e.g. islet alone group), is given by $2^{-\Delta\Delta Ct}$, where $\Delta Ct = Ct_{target\ gene} - Ct_{normalisation\ gene}$ and $\Delta\Delta Ct = \Delta Ct_{Sample} - \Delta Ct_{Calibrator}$.

## References


[1] Vaes B, Walbers S, Gijbels K, et al. (2015) Culturing protocols for human multipotent adult stem cells. Methods Mol Biol 1235: 49-58

[2] Baeke F, Van Belle TL, Takiishi T, et al. (2012) Low doses of anti-CD3, ciclosporin A and the vitamin D analogue, TX527, synergise to delay recurrence of autoimmune diabetes in an islet-transplanted NOD mouse model of diabetes. Diabetologia 55: 2723-2732

[3] Ding L, Heremans Y, Pipeleers D, et al. (2015) Clinical Immunosuppressants Inhibit Inflammatory, Proliferative, and Reprogramming Potential, But Not Angiogenesis of Human Pancreatic Duct Cells. Cell Transplant 24: 1585-1598

**ESM table 1**: Primer and probe characteristics

| Target | Sequence |
|---|---|
| **insulin** | F 5’-CCGGGAGCAGGTGACCTT-3’<br>R 5’-GATCTACAATGCCACGCTTCTG-3’<br>P 5’-AGACCTTGGCACTGGAGGTGGCC-3’ |
| **glucagon** | F 5’-AACAACATTGCCAAACGTCA-3’<br>R 5’-TGGTGCTCATCTCGTCAGAG-3’<br>SYBR |
| **somatostatin** | F 5’-GGAAACAGGAACTGGCCAAGT-3’<br>R 5’-GGGTTCGAGTTGGCAGACC-3’<br>SYBR |
| **CD31/PECAM-1** | F 5’-GCATGTCTTTTATGATCTCAGAC-3’<br>R 5’-CATCGGCAAAGTGGTCAAGA-3’<br>SYBR |

F: forward; R: reverse; P; probe; SYBR: Fast SYBR® Green

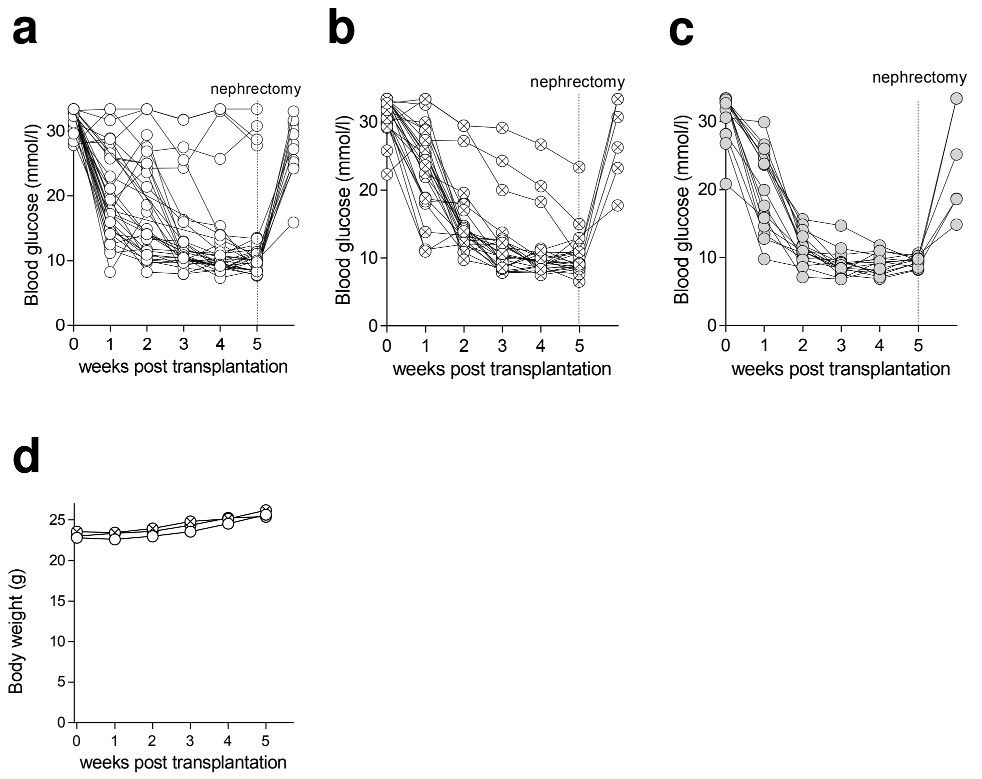


**ESM Figure 1**: Non-fasting glycaemia and body weight of transplant recipients. (**a-c**) Blood glucose concentrations were monitored in alloxan-induced diabetic C57BL/6 mice transplanted with 150 syngeneic islets either alone (**a**) (control (n=47), white circles) or co-transplanted with $2.5 \times 10^5$ human MAPC for over 5 weeks (**b-c**) (SEP, crossed circles (n=52) and MIX, light grey circles (n=40)). Recovery nephrectomies performed in randomly selected animals of each group at 5 weeks post-transplant resulted in 100% return to hyperglycaemia. (**d**) Body weight changes did not significantly differ between the various groups throughout the study period. Each value represents the mean ± SEM.

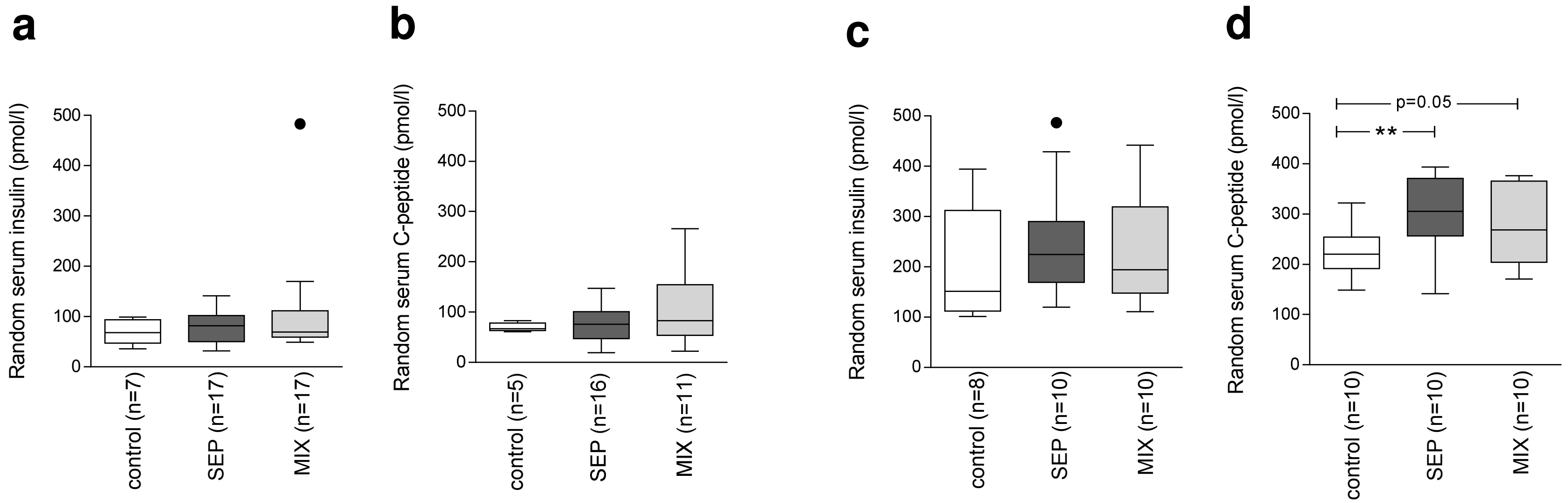


**ESM Figure 2**: Serum insulin and C-peptide concentrations 2 (**a-b**) and 5 weeks (**c-d**) post-transplantation. Mice were transplanted with 150 islets alone (control, white bars) or with 150 islets together with human MAPC as separate pellets (SEP, dark grey bars) or as a composite pellet (MIX, light grey bars). ** $p < 0.01$ versus islet-alone group (control). Solid black dots represent Tukey outliers.

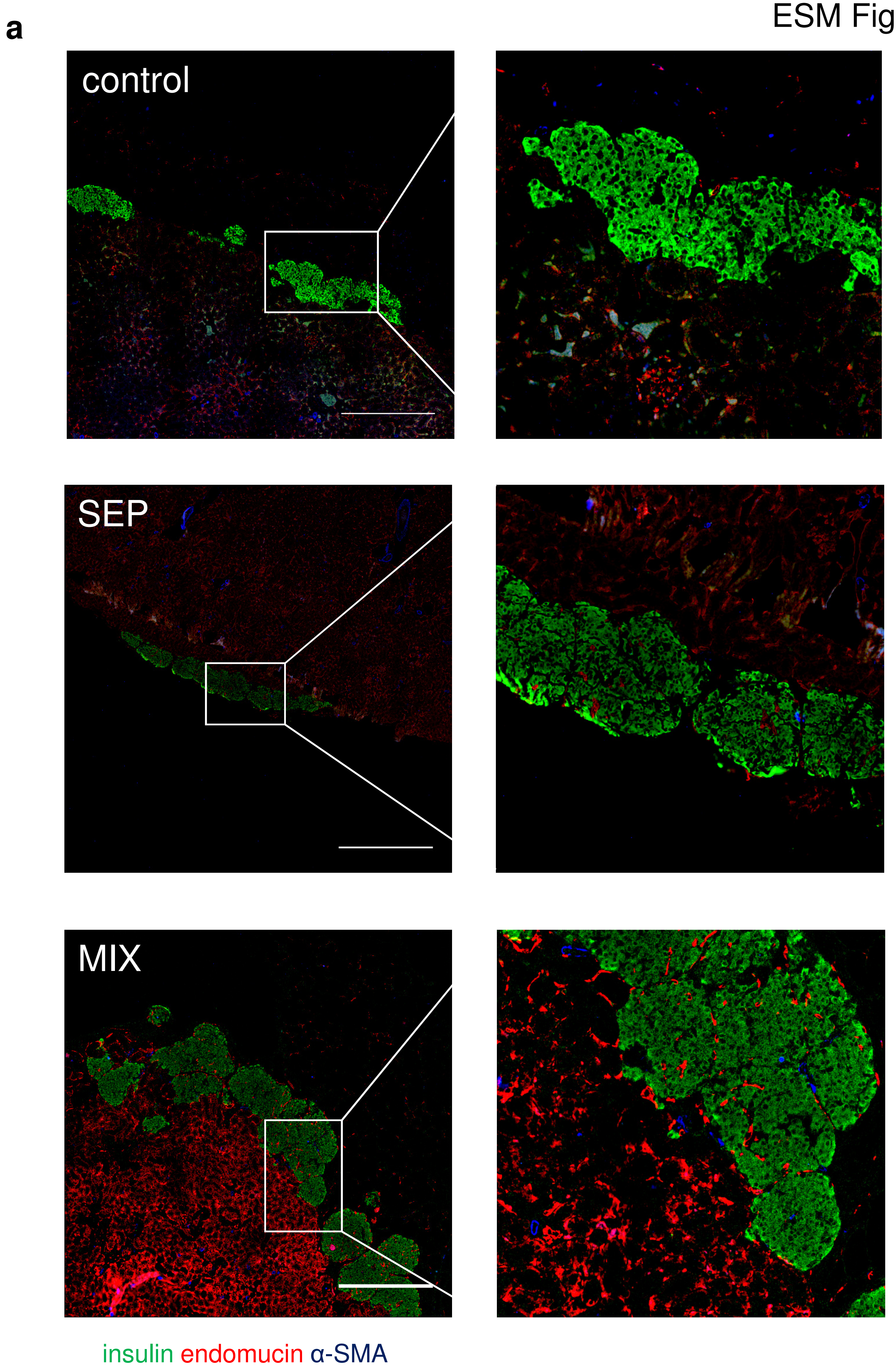
a
control
SEP
MIX
insulin endomucin α-SMA

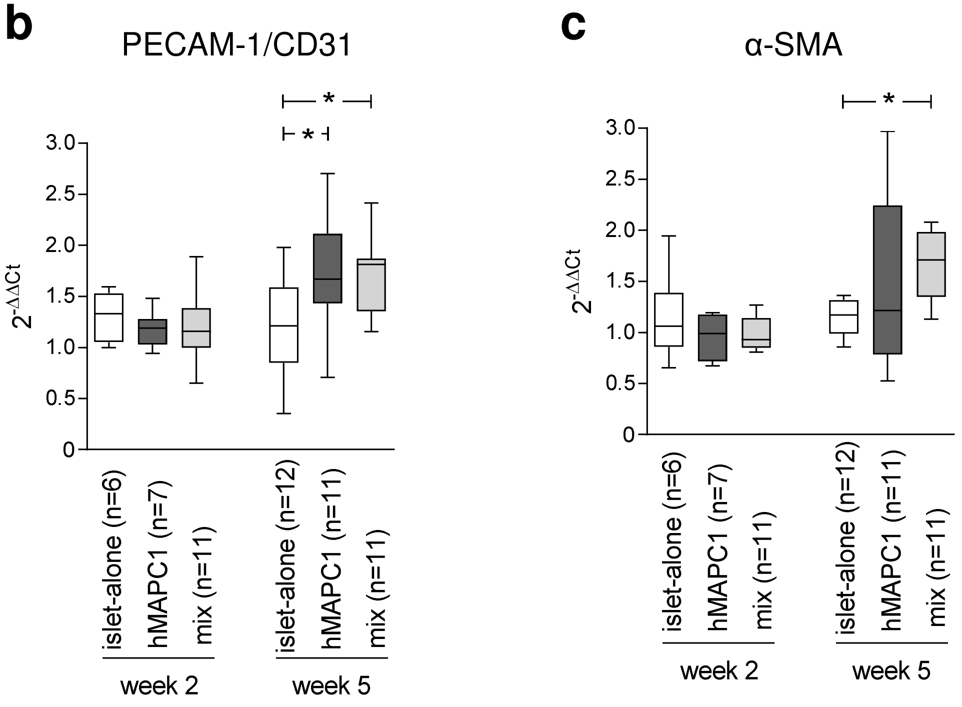


**ESM Figure 3**: Human MAPC influence islet graft revascularization under kidney capsule 5 weeks post-transplantation. (**a**) Distribution of mouse insulin- (green), endomucin- (red), and α-SMA (blue)-positive cells in islet grafts composed of islet-human MAPC as separate (SEP) or composite (MIX) pellets or of islets alone (control) at 5 weeks post-transplantation. Stitched composite images are representative of sections from 4-8 different animals. Scale bar is 500 µm. Higher magnification of the boxed area in the MIX group shows a high number of endomucin-positive endothelial cells with very few α-SMA-positive cells that encircle a well-defined lumen with fenestrations towards the adjacent endocrine cells, indicative of a new capillary-like network. This aspect may support the advanced functionality of the graft. (**b-c**) Box and whiskers plots of mRNA levels of mouse CD31/PECAM-1 (**b**) and α-SMA (**c**) in isolated islet grafts. Data are expressed as $2^{-\Delta\Delta Ct}$ (see ESM Methods). Statistical analysis was calculated using Mann-Whitney t-tests. * $p < 0.05$.
